# Resilience, Racial Identity, and Memory in Marginalized Caregiving Grandparents: The Role of Physical Activity

**DOI:** 10.1093/geroni/igaf122.889

**Published:** 2025-12-31

**Authors:** Maia McLin, Danielle Nadorff, Laura Shillingsburg, Amara Mason

**Affiliations:** Mississippi State University, Starkville, Mississippi, United States; Mississippi State University, Starkville, Mississippi, United States; Mississippi State University, Starkville, Mississippi, United States; Mississippi State University, Starkville, Mississippi, United States

## Abstract

Prospective memory failures in aging populations may reflect reduced engagement in protective behaviors like physical activity. Ethno-racially marginalized caregiving grandparents face intersecting stressors exacerbating memory challenges. While resilience and ethnic identity may buffer effects, their links to prospective memory remain underexplored. This study examines: 1) resilience-prospective memory links, 2) ethnic identity (resolution/exploration) moderation of resilience-physical activity pathways, and 3) physical activity’s mediation. A nationwide sample of 84 Black/Hispanic/multiracial grandparents (ages 45-54) completed surveys assessing resilience (MIIRM), prospective memory (PMQ; higher scores = more failures), ethnic identity (EIS_R resolution, EIS_E exploration), and physical activity. Moderated mediation analyses revealed resilience predicted more frequent memory failures (PMQ: b=0.52, p<.001), potentially explained by cognitive load theory (heightened responsibility increasing self-reported lapses). Physical activity mediated this link inversely (b = 2.86, p<.001): resilience increased activity, reducing failures among those with stronger ethnic identity resolution (B = 0.04, p<.01), aligning with social identity theory. Exploration (EIS_E) showed weaker moderation (B = 0.017, p=.023), with non-significant mediation, suggesting identity exploration offers fewer stabilizing benefits. Moderated mediation was significant for resolution (index=0.12, 95%CI[0.047,0.23]). Interventions fostering resilience and ethnic identity commitment may mitigate structural inequities’ cognitive toll through protective behaviors.

